# Effectiveness of a youth-led early childhood care and education programme in rural Pakistan: A cluster-randomised controlled trial

**DOI:** 10.1371/journal.pone.0208335

**Published:** 2018-12-19

**Authors:** Aisha K. Yousafzai, Muneera A. Rasheed, Arjumand Rizvi, Fariha Shaheen, Liliana A. Ponguta, Chin R. Reyes

**Affiliations:** 1 Department of Global Health and Population, Harvard TH Chan School of Public Health, Harvard University, Boston, United States of America; 2 Department of Paediatrics and Child Health, Aga Khan University, Karachi, Pakistan; 3 The Yale Child Study Center, Yale University, New Haven, United States of America; Australian National University, AUSTRALIA

## Abstract

**Background:**

The United Nation’s Sustainable Development Goals encompass lifelong learning from birth to youth to adulthood (Goal 4) and economic opportunities for young people (Goal 8). The targets include improving access to quality early childhood care and education (ECCE) as well as learning and training opportunities for adolescents and youth. Cross-generational models for young children and youth may offer opportunities to address the interconnections between goals and targets for the next generation. We investigated whether an ECCE programme for young children (3.5–6.5 years) delivered by female youth (18–24 years) in rural Pakistan would be effective on children’s school readiness.

**Methods:**

In partnership with the National Commission for Human Development in Pakistan, we implemented the ‘Youth Leaders for Early Childhood Assuring Children are Prepared for School’ (LEAPS) programme to train female youth to deliver ECCE. The effectiveness of the LEAPS programme on children’s school readiness was evaluated in a cluster-randomised controlled trial. We randomly allocated five clusters (villages) to receive the intervention (n = 170 children) and five clusters to control (n = 170 children). Children’s school readiness was assessed after nine months of intervention exposure using the International Development and Early Learning Assessment tool. Analyses was by intention-to-treat. The trial is registered with ClinicalTrials.gov, number NCT02645162.

**Findings:**

At endline, the intervention group had significantly higher school readiness scores (n = 166, mean percentage score 59.4, 95% CI 52.7 to 66.2) compared with the control group (n = 168, mean percentage score 45.5, 95% CI 38.8 to 52.3). The effect size (Cohen’s *d*) was 0.3.

**Conclusion:**

Trained female youth delivered an ECCE programme that was effective in benefitting young children’s school readiness. The cross-generational model is a promising approach to support early child development; however, further evaluation of the model is needed to assess the specific benefits to youth including their skills and economic development.

## Introduction

Increased investment in programmes for young children, adolescents and youth are essential to prepare the next generation with the knowledge and skills to actively participate and contribute to social and economic development in their communities [1]. Current access to learning and development programmes for this population are poor: Close to 250 million children are failing to meet their developmental potential in the first five years of life increasing risks for poor educational achievement [2]; inequalities in access to education persist in adolescence (10–19 years) and youth (15–24 years) [3]; and global unemployment rates for youth are estimated to be three times higher than for adults [3]. These challenges are recognized in the United Nation’s Sustainable Development Goals that include targets for lifelong learning from birth to youth to adulthood (Goal 4) and economic opportunities for youth (Goal 8). The interconnections between goals and targets for young children and youth offer an opportunity to examine the potential synergies and impacts of cross-generational strategies, which refer to policies and programmes intended to support more than one generation [4]. Specific targets under Goal 4 include increasing access to early childhood care and education (ECCE) programmes for young children, educational and professional opportunities for youth and the supply of qualified teachers. Informed by developmental science, a cross-generational approach may benefit these interconnected targets.

The sensitive period of brain development in early childhood is a crucial window of opportunity to mitigate risks for poor development and establish a strong foundation for lifelong health, learning and behaviour [5]. A second window of opportunity to support development is during late adolescence when more complex cognitive and social-emotional functioning emerges [6], which continues in the youth period transitioning into early adulthood [7]. In both of these sensitive periods of development, there is growth in executive functioning skills, which are cognitive processes governed by activity in the prefrontal context of the brain, related to goal-oriented behaviours such as self-regulation, reasoning skills and other higher-order thinking skills [8]. Child executive functioning is hypothesized to develop through social-interactions with others possessing strong executive functioning skills; therefore, caregivers with opportunities to develop their own executive functioning capacity are in turn more capable of supporting young children with skills development [9]. Cross-generational interventions targeting executive functioning skills may bolster gains in early childhood thereby supporting the subsequent youth transition into adulthood, and strengthen nurturing care for young children through the enhanced cognitive and social-emotional readiness of the next generation of caregivers.

Interventions that promote young children’s skills development include high quality ECCE programmes; however, data from the fourth round of the UNICEF Multiple Index Cluster Surveys show disproportionate rates of access to formal ECCE -programmes for three and four-year olds ranging from 17.8% in countries with a low human development index ranking to 50.7% in countries with a high human development index ranking [10]. Moreover, quality in ECCE programmes varies with low quality programmes less likely to support the skills young children need to be ready for school [11]. A critical supply-side barrier to participation in ECCE programmes is an inadequate number of skilled ECCE providers. Interventions to improve ECCE quality through the provision of teacher professional development benefits the structural (e.g., availability of a variety of learning resources) and the process (e.g., teacher and child interactions) quality of the programme [12].

While, youth-led models for ECCE programmes are rare, in recent years there has been an increase in youth-led programmes more generally in health and education through same-age peer mentoring and cross-age (working with younger mentees) mentoring to promote low risk behaviours and support educational activities [13]. Important characteristics for youth-led programmes include intention to benefit the youth leaders (e.g. skills development and economic opportunities), leadership skills, mentoring skills and access to mentoring for personal and programme support. Proponents of such programmes recognize youth as important agents of social awareness and transformation, and cite higher levels of creativity and energy among youth and a higher potential to introduce innovations compared to adults. With respect to ECCE, opportunities for youth educators to develop their own executive functioning capacity during a sensitive window of developmental growth may in turn support capabilities in young children with their skills development through higher quality interactions.

In countries such as Pakistan, where youth comprise 33% of the working age workforce, youth programmes that offer opportunities for education and training are in high demand [14]. The National Commission for Human Development (NCHD), Government of Pakistan, has a remit to support health and education needs in local communities, and to provide youth vocational training and economic development programmes. Vocational training for youth to deliver a high quality ECCE programme may serve as a strategy to address current gaps in the implementation of early childhood services in Pakistan. Although there is national policy support for ECCE in Pakistan, which recognizes the need to invest in improving both access and quality, its implementation is weak [15]. Quality refers both to structural quality of ECCE programmes (e.g., learning resources, teacher-child ratio) and process quality (teacher-child interactions). The *kaachi* class is the ECCE programme for young children that is available in some government primary schools. Participation in ECCE is low in Pakistan with only 23.7% of four year olds enrolled in a formal ECCE service while 25.1% are enrolled in primary school, and 51.2% are out of school [16]. Few government primary schools have dedicated space, resources, or trained teachers for the preprimary age group and studies have shown perceptions of poor quality and learning hinder participation (Rasheed et al., submitted). Programmes such as the ‘Early Learning Partnership’ implemented by the World Bank and partners in Pakistan recognize the need to improve quality for ECCE through the education system and will likely inform evidence-based strategies to improve programme quality in the Pakistani context (http://www.worldbank.org/en/topic/education/brief/early-learning-partnership). High quality ECCE prepares young children for future learning, which is critical in Pakistan where one in six children never progress from first grade in primary school [17]. Early learning programmes implemented in low- and middle-income countries (LMIC) have been effective in promoting cognitive and psychosocial outcomes [18] Curriculum which pay attention to social-interactions as well as early academic skills (numeracy in particular) with a supportive caregiver will likely support skills for learning and child executive functions. The aim of this study was to assess the efficacy of a youth-led ECCE programme for children aged 3.5 to 6.5 years old. The primary endpoint was school readiness indexed by learning, social and motor development, and executive functioning skills.

## Materials and methods

### Study design and participants

The efficacy of the ‘Youth Leaders for Early Childhood Assuring Children are Prepared for School’ (LEAPS) ECCE programme was evaluated by a cluster-randomised controlled trial in the Naushero Feroze district of Sindh, Pakistan, between November 1, 2015 and September 30, 2016. The cluster approach was used to reduce the risk of contamination by ensuring that all members of each cluster received the same intervention. The unit of cluster was the village, and we allocated the same number of clusters to each arm of the trial. On average, each cluster contained 44 children between 3.5–5.5 years old who met the eligibility criteria for ECCE enrolment. Children in the intervention group (n = 170) received ECCE through the LEAPS programme and the control group (n = 170) had access to usual educational services provided by the government. The comparisons of interest were the efficacy between the intervention and control groups on school readiness indexed by learning, social and motor development, executive functioning and participation in educational services.

In the intervention group, children aged 3.5–5.5 years in January 2016, residing in the study cluster, and enrolled in the LEAPS programme (defined by registration for enrolment at the LEAPS centre having attended at least three days of the programme in the first week) were eligible for study enrolment. In the control group, children aged 3.5 years-5.5 years, residing in the study cluster, and not attending primary school (irrespective of their ECCE enrolment status) were eligible for study enrolment. A control child could have been enrolled in any formal or informal ECCE programme (e.g., a *Kaachi* class in a government primary school, that is, a preprimary section attached to a primary school typically with no formal early childhood education curriculum). All primary caregivers provided written informed consent (or a thumb print for consent) and could refuse an interview or child assessment at any time. Ethics approval was obtained from the ethical review committee of the Aga Khan University in Pakistan.

### Randomisation and masking

**Fig 1:**
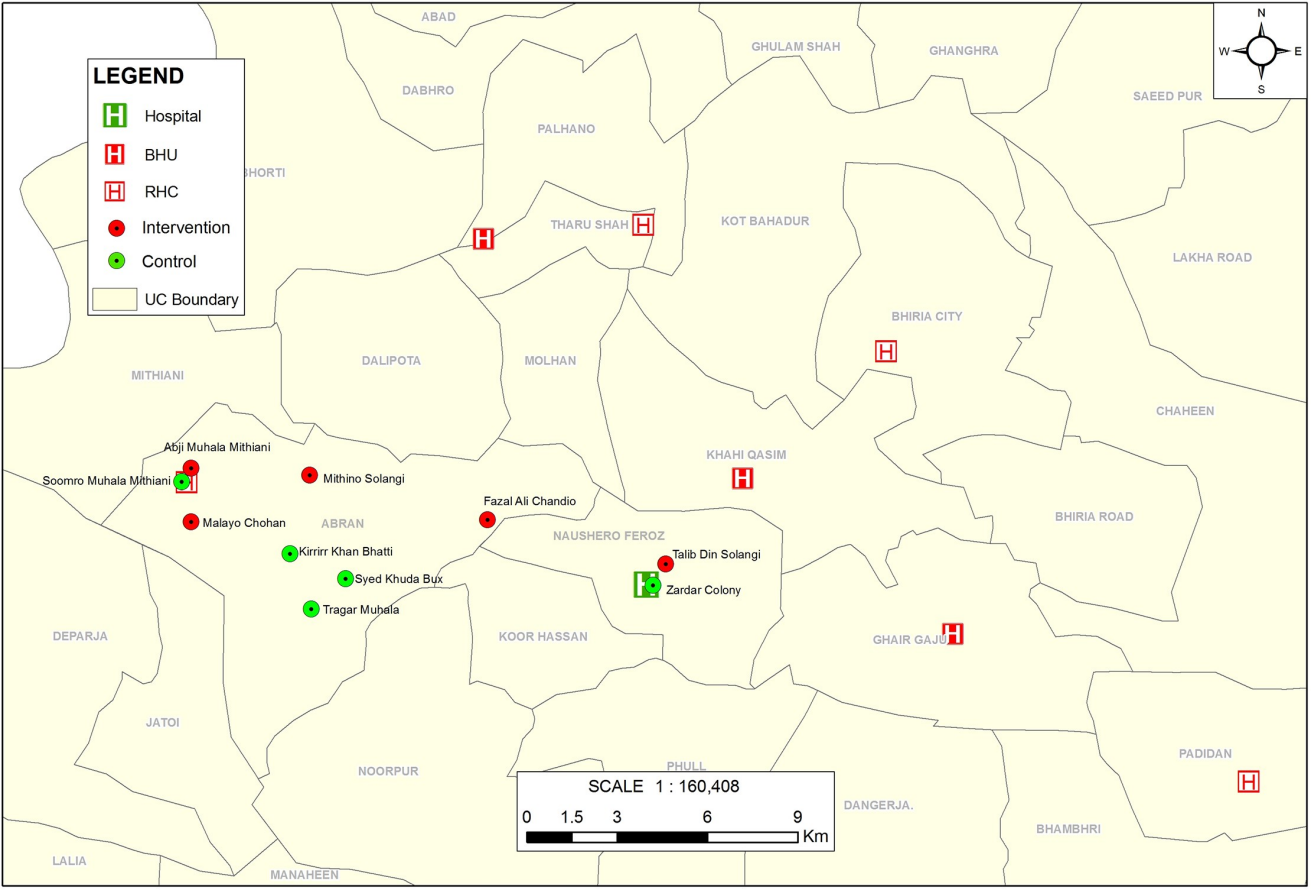
Intervention and control clusters. Five clusters were randomly assigned to the intervention arm and five clusters to the control arm for the trial.

Prior to the start of the study, we selected one Union Council (an administrative unit within a district) to take samples from, taking into consideration transport costs and travel time to ensure efficient and timely data collection visits. All villages (clusters) in the Union Council with a functional primary school were eligible for selection. Ten clusters were randomly sampled, stratified by rural or peri-urban location. Using simple randomisation, five clusters were assigned to the intervention group and five clusters were assigned to the control group (Fig 1).

The data collection team, comprising four data collectors, four community-based child development assessors and a field supervisor, were independent of the intervention team. Masking was not possible because as part of the community-based strategy, the LEAPs ECCE centres were signposted in villages and some data collection visits took place near to the centres. Quality assurance in data collection strategies to ensure precision in data collection included twice monthly field observations of each member of the data collection team, a weekly data collection team meeting, ten percent inter-observer reliability for child anthropometry measurement, ten percent video-recorded child development assessments for independent review and a refresher training prior to endline data collection.

### Intervention

The LEAPS ECCE programme was designed to address access and quality gaps to ECCE. In January 2016, nine preschools were implemented at village level and run by a Community Youth Leader (CYL). The space was provided free-of-cost by the local community; however, basic furnishing and learning resources were provided by the LEAPS programme. The CYL ran two classes per day for five days per week. The class duration was for three hours. The morning class was for younger children, aged 3.5 years to 4.5 years at the time of enrolment, and the afternoon class was for older children, aged 4.6 years to 5.5 years at the time of enrolment. The CYL-to-child ratio in each class was not expected to exceed 1:20 with remaining children placed on a waiting list. Family members and volunteers from the community were invited to spend time in the LEAPS ECCE centre to support young children or to assist the CYL in preschool organization activities (e.g., ensuring the water coolers were filled).

The LEAPS ECCE curriculum is a new curriculum developed following formative research, based on the pedagogy of the High Scope early childhood education approach and aligned with the Government of Pakistan’s early childhood education curriculum (not currently implemented in *Kaachi* classes). The seven learning areas in the curriculum were: (I) Approaches to Learning; (II) Social-Emotional Development; (III) Physical Development and Health; (IV) Language, Literacy and Communication; (V) Mathematics; (VI) Creative Arts; and (VII) My World. Community Youth Leaders supported family and community-engagement strategies by holding quarterly parent meetings, monthly meetings with the village community health worker and the local primary school teacher, and sharing information about her LEAPS ECCE centre with the NCHD and district officials in quarterly town hall meetings.

The CYL recruitment strategy was implemented in partnership with the NCHD and village stakeholders. First, community meetings were conducted with village leaders to identify eligible candidates (women with at least 10 years of education, aged 18–24 years, and residing in the same village). The LEAPS programme was described and the village leaders identified young women in their community who met the age and education criteria. Eighteen women were nominated and were met by programme staff to discuss the LEAPS programme and to ascertain their interest. Twelve short-listed candidates participated in a one-day workshop to evaluate their potential to be ECCE providers. Activities were organized to assess literacy, numeracy and general knowledge, creativity, problem solving, planning and leadership skills. Following the workshop, eleven candidates were selected to participate in the one-month basic training and at the end of this training 10 CYLs were recruited. One CYL was an Associate CYL who supported LEAPS ECCE centres if the regular CYL was unwell or on leave. Among the 10 CYLs, three youth leaders were married and only one had a child.

The one-month basic training for CYLs comprised centre-based learning and teaching practice sessions. The training imparted knowledge (e.g., principles of ECCE), promoted positive attitudes (e.g., respect for young children, responsibility, cooperation, confidence-building), and skills (e.g. executive functioning skills through activities building abilities to organize, plan, coordinate, and critically reflect, responsive interactions to engage with young children and scaffolding to support learning). Following training, the CYLs received at least twice monthly support from a Mentor and participated in Communities of Practice (CoP) meetings held weekly in the first month, fortnightly in the second month, and then monthly. The goal of the professional development support was to provide additional guidance on ECCE service provision, review curriculum planning, enable peer-to-peer learning to share success stories and solve problems, and offer career guidance. The CYLs received a monthly stipend of Pakistani Rupees 5000 (USD 50) aligned with the NCHD youth programmes. The stipend aligns with the amount stipulated by the NCHD that was provided for youth vocational training programmes at the time of the study. The stipend of Pk Rs 5000 per month is lower than the salary of NCHD feeder school teachers (approximately Pk Rs 8000 per month) and government primary school teachers (approximately Pk Rs 4000 to Pk Rs 16 0000 per month). It is better than the salary provided to some private providers of preschool services (although these are less common in rural settings). In addition to the stipend, transport support was offered to CYLs to participate in the training and CoP because the lack of public transport in rural areas and prevailing social norms about travelling alone were barriers for participation among young women in this rural community. The CYLs received a certificate after successfully completing the one-month basic training and six-months of supervised on-the-job training.

### Data collection

**Fig 2:**
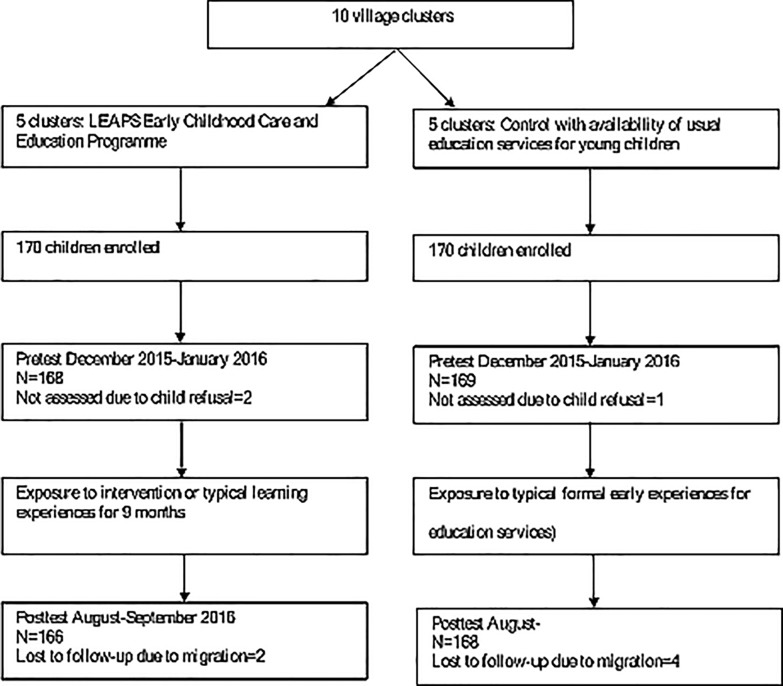
Flowchart of clusters, participants, and timeline.

All questionnaires and child assessments were administered in Sindhi. We followed language and sociocultural adaptation protocols to ensure that the conceptual integrity of the original items was retained in adaptation [19]. Data were collected pre-intervention (December 2015-February 2016) and after nine-months of intervention exposure (August-September 2016) in the intervention and control groups (Fig 2). Data were collected at the child’s home. The field supervisor observed approximately 10% of assessments for reporting inter-observer reliability.

Children’s school readiness was assessed using the International Development and Early Learning Assessment (IDELA) tool developed by Save the Children and previously tested and validated in Pakistan (www.savethechildren.org) and an executive functioning battery. The IDELA measures children’s learning, social-emotional, and motor development. Internal consistency was good (Cronbach’s α = 0.993) and inter-observer reliability between the supervisor and child development assessors was high (n = 54, R = 0.987, p<0.0001). A battery of tasks was created to assess children’s executive functioning following the same adaptation protocols previously used for a similar age-group in Pakistan [20]. The *Dimensional Change Card Sort* (DCCS) is a cognitive flexibility task that measures children’s ability to switch between two different dimensions and to think about multiple dimensions simultaneously [21]. Children are requested to sort cards at three levels. In the first level, the separated version of the DCCS, the cards depict a black silhouette shape (e.g., rabbit or boat) on a colored card (e.g., red or blue). Thus, the shape and color dimensions are separated. In the second level, the shape and color are integrated on the stimuli card (i.e., red rabbit and blue boat on a white card). In the third level, the integrated pictures from the previous level are used, but half of the cards have borders. On the border card, the child sorts by colour, and on a card without a border, the child sorts by shape. The DCCS showed good internal consistency (Cronbach’s α = 0.059) and inter-observer reliability between the supervisor and child development assessors was high (n = 63, R = 0.951, p<0.0001). *Knock Tap* and *Peg Tap* assess inhibitory control [22]. In Knock Tap, the child is required to knock with their knuckles on the table or tap with the flat of their palm in the opposite action to that of the assessor (i.e., child knocks when the assessor taps). In Peg Tap, the child has to knock once if the assessor knocks twice or knock twice if the assessor knocks once. Knock Tap and Peg Tap had good internal consistency (Knock Tap Cronbach’s α = 0.934 and Peg Tap Cronbach’s α = 0.991) and inter-observer reliability between the supervisor and child development assessors was high (Knock Tap n = 62, R = 0.868, p<0.0001 and Peg Tap n = 62, R = 0.978, p<0.0001). The *Corsi Blocks* task assesses working memory and requires the child to tap the blocks in a sequence after observing the assessor tap a sequence on a set of nine blocks [23]. This task had good internal consistency (Cronbach’s α = 0.963) and inter-observer reliability between the supervisor and child development assessors was high (n = 63, R = 0.915, p<0.001). A secondary outcome was a 15-item child report on their health, hygiene and nutrition knowledge and practices.

Potential confounding variables were collected including: (1) caregiver reports on ECCE exposure pre- and post-intervention (verified by administrative records, but this was not possible in control groups); (2) parental education and employment, household socio-economic status and food security information pre-intervention; and (3) Child IQ using the performance subscales of Weshler Preschool and Primary Scales of Intelligence, Third Edition [24]. previously adapted and tested for use in a rural Pakistani population [25]. Performance IQ was derived from 3 subtests of the WPPSI III for children older than 4 years and from 2 subtests for children younger than 4 years as per the WPPSI III protocol. Children’s height and weight were measured pre-intervention according to standard protocols [26]. Height (Shorr Board, Weigh and Measure LLC, USA) was measured to the nearest 0.1cm and weight (Seca 877 Digital Flat Scale, Weigh and Measure LLC, USA) was measured to the nearest 0.1kg, and scales were calibrated each morning before data collection visits with standard weights. The relative technical error of measurement (TEM) was good for anthropometric measures assessed in children (height n = 33, TEM 0.038, R = 0.999; weight n = 33, TEM 0.856, R = 0.999).

### Statistical analyses

We used PASS (version 11) to establish the sample size. The required sample size was estimated based on a preschool study conducted in Bangladesh [27, 28]. Intra-cluster correlation was 0.14 was calculated using maternal education data for the study area [29]. A sample size of 30 children per cluster was estimated at 90% power and α of 0.05 to detect differences of at least 4 SD for reading adjusted for a 10% attrition rate. Mixed effect linear regression was used to model linear outcome whereas mixed effect binary regression was used for the binary coded outcomes. The model incorporated cluster as random intercept to account between cluster variability. Fixed effect included time variable (duration between baseline and endline assessments), main effect of group and a time x group interaction term to examine the effect of intervention with time. Significance was defined as a p value lower than 0.05 unless otherwise stated. Baseline differences between groups were tested to identify potential confounders that would need to be accounted for in the analyses of the outcome variables. Group differences in child outcomes comparing the intervention and control groups were then assessed. Finally Cohen’s *d* effect sizes were calculated as differences in adjusted means between the intervention and control group over the pooled SD. Stata version 12.1 was used to conduct all statistical analyses. The original trial is registered with ClinicalTrials.gov, number NCT02645162.

### Role of the funding source

The funders had no role in the study design, data collection, data analyses, data interpretation, or writing of the report. The authors of this report had full access to all data in the study. AKY had primary responsibility to submit the manuscript.

## Results

The intervention was implemented as intended with respect to training and mentoring contacts for the CYLs. Documentation was maintained in training attendance, pre- and post-training assessment records, mentoring checklists and records of CoP meetings. Two preschool classes were run as intended per day from Monday to Friday for a three hour session. Data and information sought from the Mentoring Checklists and the CoP meetings informed real-time programme quality improvements. Key strategies for the improvement of programme quality were scaffolding skills of the CYLs during mentoring contacts and in the CoP meetings, and sensitization strategies with caregivers to instill the importance of regular and on-time attendance of children in the LEAPS ECCE programme.

A total of 340 children were enrolled in the study (n = 170 intervention group, n = 170 for the control group). Baseline characteristics of the intervention and control group are shown in Table 1. There were no significant differences in baseline characteristics between the six children lost to follow-up and the sample follow-up. The intervention and control group characteristics were comparable at baseline. No significant differences in child, parental and household baseline characteristics between the intervention groups except for the ECCE enrolment status (Intervention group 100% *Vs*. Control group 40.6%, p<0.0001).

**Table 1:** Study population characteristics at enrolment.

	Intervention Group N = 170	Control Group N = 170	p Value
**Child Characteristics**			
Age, months	55.2 (7.6) 95% CI 54.04, 56.33	54.3 (7.8) 95% CI 53.13, 55.5	0.292
Girls, %	49.4	48.8	0.900
Birth order	3.4 (1.2) 95% CI 3.04, 3.71	3.3 (4.2) 95% CI 3.0, 3.61	0.834
Height-for-Age Z score	-1.89 (1.0) 95% CI -1.79, -1.48	-1.64 (2.15) 95% CI -2.04, -1.73	0.171
Weight-for-age Z score	-1.42 (1.14) 95% CI -1.55, -1.29	-1.33 (1.13) 95% CI -1.46, -1.20	0.471
Child IQ (Performance IQ)	77.6 (9.3) 95% CI 76.1, 78.9	75.4 (9.3) 95% CI 73.9, 76.9	0.192
Preschool enrolment, %	100	40.6	<0.0001
**Parental Characteristics**			
Maternal literacy, %	31.8	39.4	0.566
Maternal employment, %	30.0	18.2	0.223
Paternal literacy, %	77	69	0.218
Paternal employment, %	97.6	97.6	0.979
**Household Characteristics**			
Socio-economic score	-0.15 (2.48) 95% CI -0.29, -0.14	0.15 (4.17) 95% CI -0.008, 0.31	0.418
Food insecure, %	30.6	24.7	0.225

Mean (SD) reported unless otherwise stated. Child IQ assessed using the Performance IQ scale of the Weshler Preprimary and Primary Scales of Intelligence, Third Edition.

In the intervention group, study children all met the enrolment criteria for the preschool placement and represent 71.1% of total number of children enrolled in the LEAPS ECCE programme (n = 170/239) with 45.6% enrolled in the morning classes for younger children and 54.4% enrolled in the afternoon classes for older children. The proportion of total girls’ enrollment was 44.7%. In the control study group, 40.6% of children were enrolled in any ECCE service (n = 69) with 42 children reported to have less than six months of ECCE programme exposure and 27 children ≥ six months of ECCE programme exposure. At endline there were significant differences in ECCE participation between the intervention and control group (p<0.0001): 88% of intervention group were enrolled in LEAPS ECCE (n = 146) with nearly all having attended for at least six months (n = 145) compared to the control group ECCE enrolment of 57.1% (n = 96) with close to half with less than six months exposure (n = 44) and the remainder with at least six months exposure (n = 52).

Children’s school readiness at baseline and endline comparing the intervention and control group is shown in Table 2. Data are adjusted for clusters, differences in baseline ECCE exposure, and pre-intervention assessment scores for school readiness. After nine months of intervention exposure, children in the intervention group had significantly higher school readiness score as indexed by the IDELA (n = 166, mean 59.4, 95%CI 52.7, 66.2) compared with the control group (n = 168, mean 46.0, 95%CI 38.8, 52.3, P<0.0001). The mean scores for each subtest of the IDELA for the intervention group were significantly higher than for the control group. The intervention effect size was 0.3 for the overall IDELA assessment ranging from 0.1 for fine and gross motor skills to 0.6 for socioemotional skills.

**Table 2:** Children’s school readiness pre- and post-intervention.

	Intervention Group		Control Group		p Value	Cohen’s *d*
	Pretest N = 170	Posttest N = 166	Pretest N = 170	Posttest N = 168		
**IDELA**	**Mean, 95% CI**					
Total Score	35.8 (29.0, 42.5)	59.4 (52.7, 66.2)	40.0 (33.2, 46.7)	46.0 (38.8, 52.3)	<0.0001	0.31
Emergent mathematics subtest	12.5 (11.0, 14.0)	16.6 (15.2, 18.1)	12.2 (10.7, 13, 7)	13.8 (12.3, 15.3)	0.001	0.29
Socioemotional subtest	7.3 (6.1, 8.6)	12.5 (11.2, 13.9)	8.0 (6.8, 9.3)	7.6 (6.3, 8.8)	<0.0001	0.61
Self-regulation subtest	1.1 (0.9, 1.3)	1.9 (1.7, 2.2)	1.2 (1.0, 1.4)	1.6 (1.4, 1.8)	0.002	0.22
Emergent literacy subtest	7.0 (5.3, 8.7)	12.8 (11.1, 14.5)	8.6 (6.9, 10.3)	9.4 (7.7, 11.1)	<0.0001	0.31
Fine motor subtest	5.5 (4.2, 6.9)	8.8 (7.4, 10.1)	6.4 (5.0, 7.7)	8.0 (6.7, 9.4)	0.01	0.09
Gross motor subtest	2.3 (0.5, 4.0)*	6.8 (5.1, 8.2)	3.6 (1.8, 5.4)**	5.2 (3.5, 6.9)	0.005	0.14
**Executive Functions**	**N (Passed Practice Trial), Mean, 95% CI**					
Working memory-Corsi Blocks	129, 1.0 (0.8, 1.2)	152, 1.4 (1.2, 1.5)	117, 1.2 (1.0, 1.4)	135, 1.3 (1.1, 1.5)	0.023	0.05
Cognitive flexibility-DCCS: Pre-switch separated	113, 5.9 (5.8, 5.9)	149, 6.0 (5.9, 6.0)	92, 5.9 (5.8, 5.9)	120, 5.9 (5.9, 6.0)	0.232	0.23
Cognitive flexibility-DCCS: Post-switch separated	73, 5.8 (5.7, 5.9)	101, 5.8 (5.7, 5.9)	47, 5.8 (5.6, 5.9)	54, 5.9 (5.9, 6.0)	0.993	0.10
Cognitive flexibility-DCCS: Pre-switch integrated	71, 6.0 (5.9, 6.0)	100, 6.0 (6.0, 6.0)	39, 6.0 (6.0, 6.0)	51, 6.0 (6.0, 6.0)	0.832	0.832
Cognitive flexibility-DCCS: Post-switch integrated	16, 6.0 (5.9, 6.1)	63, 5.9 (5.8, 6.0)	12, 5.7 (5.6, 5.9)	18, 6.0 (5.9, 6.1)	0.005	-0.38
Inhibitory control-Knock Tap	151, 9.6 (8.5, 10,6)	161, 12.8 (11.8, 13.8)	127, 10.0 (8.9, 11.0)	151, 11.5 (10.4, 12.5)	0.018	0.20
Inhibitory control-Peg Tap	128, 6.7 (5.4, 7.8)	150, 10.3 (9.1, 15.8)	98, 8.6 (7.3, 9.9)	129, 9.3 (8.1, 10.5)	<0.0001	0.15

Significant differences are shown for post-intervention scores comparing mean scores for intervention and control groups adjusted for clustering, baseline preschool exposure, and baseline scores in school readiness assessments.

*N = 169

**N = 168

Abbreviations: DCCS-Dimensional Change Card Sort, IDELA-International Development and Early Learning Assessment

Children’s executive functioning abilities were higher after nine months of intervention exposure in the intervention group compared to the control group. Significant differences were found in scores for working memory (Intervention group: n = 152, mean1.4, 95%CI 1.2, 1.5 *Vs* Control group: n = 135, mean 1.3, 95%CI 1.1, 1.5, p = 0.023) and for inhibitory control measured by Knock Tap (Intervention group: n = 161, mean 12.8, 95%CI 11.8, 13.8 *Vs* Control group: n = 151, mean 11.5, 95%CI 10.4, 12.5, p = 0.018) and Peg Tap (Intervention group: n = 150, mean 10.3, 95%CI 9.1, 15.8 *Vs* Control group: n = 129, mean 9.3, 95%CI 8.1, 10.5, p<0.001). For the cognitive flexibility task, each subset of tasks is progressively more challenging with fewer children in both the intervention and control groups successfully progressing forward to the next subset. A significant difference is observed in the final subset of tasks (level three sorting cards with and without a boarder) with a slightly lower score in the intervention group compared to the control group; however, fewer children in the control group progressed to this task (Intervention group: n = 63, mean 5.9, 95%CI 5.8, 6.0 *Vs* Control group: n = 18, mean 6.0, 95%CI 5.9, 6.1, p = 0.005). A significant small intervention effect size was only found on working memory (Cohen’s *d =* 0.1) and inhibitory control (Cohen’s *d =* 0.2).

Children were also asked to report on knowledge and practices about health, hygiene and nutrition. At baseline the knowledge and practice scores between the intervention and control group were not significantly different (Intervention group: n = 170, mean 3.3, 95%CI 2.6, 4.0 *Vs* Control group: n = 170, mean 2.8, 95%CI 3.1, 3.5). Following nine months of intervention exposure, children in the intervention group had significantly higher scores than children in the control group Intervention group: n = 166, mean 5.8, 95%CI 5.1, 6.4 *Vs* Control group: n = 168, mean 3.7, 95%CI 3.0, 4.4, p<0.001).

## Discussion

This study was designed to test whether an ECCE programme delivered by CYLs in a rural setting could improve participation of children in ECCE and be effective for children’s school readiness. The intervention resulted in increased participation of young children in ECCE compared with children in the control group. Trained CYLs were delivered a programme that was effective on children’s school readiness indicted by significantly greater school readiness scores among the intervention children compared to the control children with improvements in early academic skills (e.g. emergent literacy and mathematics) as well as in socioemotional skills and executive functioning. In order to achieve this, the CYLs were trained and mentored to deliver an evidence-based ECCE curriculum with mentorship focusing on fostering responsive and socioemotionally supportive interactions as well as scaffolding learning abilities in young children. These results confirm that investment in ECCE supports children’s development and learning outcomes [18, 30]. A recent meta-analyses of early learning programmes in LMICs found that formal and non-formal or community-based preschools had a mean effect size of SMD 0.67 (95% CI 0.43–0.21) on cognitive outcomes and SMD 0.23 (95% CI0.06–0.4) on psychosocial outcomes [28]. In comparison to these meta-analyses findings, the LEAPS programme had a lower impact on cognitive or early learning outcomes (Cohen’s *d =* 0.30) and a higher impact on psychosocial outcomes (Cohen’s *d =* 0.61). The LEAPS curriculum content focus had many opportunities for supporting children’s social-emotional development to support school readiness, which may have benefitted children’s social-emotional development including activities on motivating approaches to learning, social skills and communications as well as attention to the CYL-child interaction. The review reported that non-formal or community-based preschools, such as LEAPS, typically had lower effect sizes than formal preschools. However, regardless of the model, quality in the programme mattered (e.g., variety of early learning resources, classroom organization and teacher-child interaction). The importance of attention to quality in the learning setting is noteworthy for attaining higher effects on child learning outcomes [11, 18, 30], and in the LEAPS programmes this was supported through mentorship of the CYLs.

In order for Pakistan to achieve the sustainable development goal target 4.1 (all children are able to access quality early childhood development, care and learning) substantial increases in investment will be needed for early years programmes. The current *Kaachi* class system in Pakistan (preprimary class in a primary school) is one part of the solution, but will require focus on improving access, quality and equity in the country. Intervention research from Chile demonstrate strategies that potentially support quality in the ECCE setting through provision of improved professional development opportunities [12].

Youth-led ECCE may offer an option to the range of solutions required [13]. Vocational training and skills development for youth is central to the Government of Pakistan’s NCHD, but programmes meeting the needs of rural female youth are few. The NCHD is also responsible for supporting the education sector by establishing functioning primary schools (feeder primary schools) in villages where currently there is no functioning primary school. Female youth training in ECCE linked to implementing services in villages with feeder primary schools run by the NCHD is an opportunity to meet the interconnected targets under Goal 4 of the Sustainable Development Goals [4] This efficacy trial demonstrated communities were willing to provide space and accept female CYLs delivering ECCE for their young children. Family and community engagement has been shown to be a key ingredient of young children’s successful school readiness [31]. The development of the intervention aligned with features common to the NCHD strategy for community-engagement (e.g., recruitment of CYLs), and vocational training (e.g. stipend). Taking the LEAPS model to scale will require a transition-to-scale participatory research (e.g., Plan-Do-Study-Act) that enables the government system to build capacity to take-up the intervention in a sustainable manner [32].

This study has a number of limitations. Firstly, we were unable to follow-up the children for twelve months (or one academic year); however, the results are promising for nine months of intervention exposure, which also included vacation time for religious and national holidays. Secondly, we cannot report whether the early differences in school readiness will make a difference to educational attainment when children enter primary school. Thirdly, while the evaluation team was independent of the intervention team they were not fully masked to the intervention assignment of the cluster, which was not possible due to the nature of the community-based intervention implementation strategy. Further analyses from the process evaluations will be undertaken to describe fidelity and to explore the programme implementation features in order to provide insight on feasibility, quality and demand. Limitations not-withstanding, the study has a number of strengths including a robust evaluation design and reliable assessments.

## Conclusions

Innovations and integrated planning is needed to meet the Sustainable Development Goals for young children, adolescents and youth by 2030. Multiple solutions across sectors will likely support progress for young children’s development, care and learning including improving numbers of skilled ECCE personnel in the workforce (1). Further analyses is underway to report on the intervention benefits to youth themselves. This intervention shows promise that youth can be effective transformative change agents in communities to achieve these goals as well as beneficiaries. With regards to generalizability, the youth-led model may be particularly relevant in contexts like Pakistan with high youth populations, and especially limited economic opportunities for female youth.

## Supporting information

S1 File Study data. (XLSX)
